# 525. Comparison of the acceptability and safety of molnupiravir in COVID-19 patients aged over and under 80 years

**DOI:** 10.1093/ofid/ofad500.594

**Published:** 2023-11-27

**Authors:** Kohei Fujita, Osamu Kanai, Hiroaki Hata, Takao Odagaki, Tadashi Mio

**Affiliations:** National Hospital Organization Kyoto Medical Center, Kyoto, Kyoto, Japan; National Hospital Organization Kyoto Medical Center, Kyoto, Kyoto, Japan; National Hospital Organization Kyoto Medical Center, Kyoto, Kyoto, Japan; National Hospital Organization Kyoto Medical Center, Kyoto, Kyoto, Japan; National Hospital Organization Kyoto Medical Center, Kyoto, Kyoto, Japan

## Abstract

**Background:**

Molnupiravir is being widely used as a treatment for coronavirus disease 2019 (COVID-19); however, its acceptability and safety in older patients aged ≥ 80 years in real-world clinical practice is not well understood.

**Methods:**

We conducted a single-centre retrospective study and assessed the outcome of patients with COVID-19 treated with molnupiravir according to the following criteria: (A) discontinuation rate of molnupiravir; (B) type, frequency, and severity of adverse events; (C) all-cause mortality within 30 days of the diagnosis of COVID-19.

**Results:**

Forty-seven patients (46.1%) were aged ≥ 80 years (older patients) and 55 (53.9%) were aged < 80 years (younger patients). There were no significant differences in coexisting diseases and history of vaccination for COVID-19 between older and younger patients. Older patients were significantly more likely to have moderate disease (moderate 1 and 2) according to the Japanese Ministry of Health, Labour and Welfare classification than younger patients. During treatment, 8.5% of older patients and 1.8% of younger patients stopped taking molnupiravir, but the difference was not significant. Adverse events were observed in 39/102 (38.2%) patients. The most common adverse events were diarrhoea (9.8%), exacerbation of coexisting diseases (6.9%), bone marrow suppression (6.9%), liver dysfunction (5.9%), and loss of appetite (4.9%). Most adverse events were minor, ranging from grades 1 to 3. The all-cause mortality rate was 10.8%, and no molnupiravir-related deaths were observed.

**Conclusion:**

Molnupiravir treatment is acceptable and safe in older patients with COVID-19 aged ≥ 80 years.

**Disclosures:**

**All Authors**: No reported disclosures

